# Exaggerated levels of some specific TLRs, cytokines and chemokines in Japanese encephalitis infected BV2 and neuro 2A cell lines associated with worst outcome

**DOI:** 10.1186/s12985-023-01966-8

**Published:** 2023-01-27

**Authors:** Mukti Shukla, Atul Garg, T. N. Dhole, Rachna Chaturvedi

**Affiliations:** 1 Amity Institute of Biotechnology, Amity University, Lucknow Campus, Lucknow, Uttar Pradesh 226028 India; 2 Sanjay Gandhi Post-Graduate Institute of Medical Sciences, Lucknow, 226014 India

**Keywords:** JEV, TLRs, Cytokines, Chemokines, Inflammation

## Abstract

Japanese encephalitis (JE) disease, a viral brain fever is caused by Japanese encephalitis virus (JEV). Despite the availability of effective vaccines against this deadly infection, JE is the leading cause of epidemic viral encephalitis in children in South-east Asia. There is no treatment available for the JE disease which might be due to incomplete understanding of the pathogenesis of JE virus. The JEV infections lead to permanent neurological deficits even in those who survive from the infection. Activated microglia may play a potentially detrimental role by eliciting the expression of pro-inflammatory cytokines such as interleukin (IL)-1β, IL-6, and tumor necrosis factor-α (TNF-α) influencing the surrounding brain tissue. Microglial activation, proinflammatory cytokine release and leukocytes trafficking are associated following JEV infection in central nervous system (CNS). How the pattern recognition receptors sense the viral nucleic acid and how the microglial and neuronal cells behaves following JEV infection is still unelucidated. There is scarcity of data on the expression levels of toll like receptors (TLRs), cytokines and chemokines in JEV infection in invitro model. To explore the molecular mechanisms of JEV infection of microglial cells and neuronal cells, we studied the expression profile of TLRs, cytokines and chemokines in JEV infected microglial cell line BV2 and Neuronal cell line Neuro 2A. For the present study, we developed the mouse model of encephalitis by intracerebral (IC) injection of JE virus for virus propagation, disease progression and damage study. Our results demonstrate the exaggerated release of some specific TLRs, cytokines and chemokines in invitro cell culture of microglial and Neuro 2A cell line, which are associated with bad outcome in invivo study.

## Introduction

Japanese encephalitis (JE) disease is caused by Japanese encephalitis virus (JEV). JEV belongs to genus flavivirus of the family *flaviviridae*. The JEV genome has positive sense single stranded RNA which comprise of one open reading frame, encoding three structural proteins (C, prM/M, and E) and seven nonstructural proteins (NS1, NS2A, NS2B, NS3, NS4A, NS4B, and NS5). The virus is transmitted by mosquito vectors in a zoonotic cycle involving wild aquatic bird reservoirs and pigs as amplifying hosts. Humans are dead-end hosts, because low viremia does not allow further viral spread [1]. There are number of studies suggesting that in JEV infection, there is an acute and uncontrolled inflammation in the central nervous system (CNS) resulting in neuronal damage in humans, especially affecting children. However, the pathway of JEV entry into CNS and mechanisms associated to the inflammatory response are not completely elucidated. The JEV have devised several strategies to subvert the innate immune response in order to establish in host [2]. In India, thousands of children’s die due to this deadly virus each year despite the availability of vaccine. At present there is no specific treatment available for this deadly virus infection. This is due to incomplete understanding of the disease pathology. Thus, there is an urgent need to better understand the immunology of this virus in order to develop novel therapies.

JEV infection causes irreversible damage to neurons. Microglial activation can lead to bystander damage to the neuronal cells possibly through releasing cytokines and chemokines. Activated microglia cells have a dual role: these cells play a beneficial role by phagocytosis of damaged tissue but also release inflammatory mediators’ tumor necrosis factor (TNF)-α, Interleukin (IL)-1β, IL-6, and IL-17, which enhance inflammation and tissue damage [3]. JEV infection is associated with microglial activation in the CNS resulting in the production of pro-inflammatory cytokines [4]. Following activation microglia express markers characteristic of M1 activation including increased expression of Ionized calcium binding adaptor molecule (Iba)-1, as well as chemokines such as C–C motif cytokine 2 (CCL2), CCL3, CCL5, and CCL7 [5].

TNF-α, can regulate the expression of chemokine/chemokine receptors and other adhesion molecules by endothelial cells of the brain and they can enhance the trafficking and the homing of inflammatory leukocytes to the brain, leading to an exacerbated disease profile [6, 7]. TNF-α signaling in microglia upon JEV infection leads to glutamate release, contributing to neuronal death [8]. Other inflammatory mediators, such as nitric oxide (NO), produced by microglia can lead to direct neuronal death [9]. Chemokines are constitutively expressed, and their expression can be increased by inflammatory mediators in a wide range of cell types and tissues, including the CNS [10]. Microglia and astrocytes were the predominant cells for JEV-induced regulated on Activation, Normal T Expressed and Secreted (RANTES) expression. Signaling pathway of JEV-induced RANTES production was associated with the activation of extracellular signal-regulated Kinase (ERK) and increased binding activity of NF-IL-6 and NF-Kβ [11]. The differential expressions of cytokines and chemokines are observed in mice brain and spleen during JEV infection [12]. Altered homeostasis of chemokines including CCL2 (also known as MCP-1 [monocyte chemoattractant protein]), CCL3 (also known as MIP-1α [macrophage inflammatory protein 1-α]), CCL5 (also known as RANTES, CXCL8 (IL-8) and cytokines viz. TNFα, IL-6, IL-1β have been associated with the disease outcome [9, 13–16]. MCP-1 levels were significantly upregulated in JEV-infected spleen in a time dependent manner [17]. In vitro studies demonstrated expression of cytokines and chemokines in neural cells after JEV infection [9].

Toll like receptors (TLRs) play a pivotal role in orchestrating immune responses [18]. There are studies which demonstrated that the activation of TLR9 signaling exacerbate neurodegeneration by inducing oxidative stress and inflammation. In vivo studies of different viral encephalitis models and in vitro exposure of primary microglia and mouse and human microglial cell lines to TLR3 ligand poly(I:C) or TMEV and cGAS ligand cyclic guanosine monophosphate–adenosine monophosphate (cGAMP) or HSV-1 showed the expression of several cytokines and chemokines including IFN-β, IFN-γ, TNF-α, IL-1β, CCL2, CCL5, and IL-6. These proinflammatory molecules induce neuronal death by causing direct and indirect neurotoxicity [9, 19, 20]. Earlier study documented that pattern recognition receptors (PRRs) appear to be involved in JEV infection in Neuro 2A cells and brains of suckling and adult mice [21]. Significant upregulation of TLR2 and MDA5 expression levels were observed in JEV infected mice and Neuro 2A cells respectively [21].

We explored the microglial activation induced cytokine, chemokine and TLRs expression in BV2 and Neuro 2A cell lines and impact of these immune mediators on the outcome of Japanese encephalitis disease.

## Materials and methods

### Virus propagation and virus titer determination for invitro study

An Indian non-neuroinvasive but neurovirulent strain of JEV, GP78668A (GP-98) was used throughout the study. The virus was propagated in the brain of suckling mice by intracerebral (IC) injection. Mice were procured and housed at an animal care facility of our Institute i.e. Sanjay Gandhi Post Graduate Institute of Medical Sciences (SGPGIMS), Lucknow, India. Mice were fed with protein rich diet and water ad libitum. The animals were maintained in an air conditioned room (25 ± 2 °C) with 12-h light and dark cycle (7 a.m.–7 p.m.). All the experiments were performed during the light cycle, between 10 a.m. and 2 p.m., and were normalized in all mice. The study was approved by the institutional ethics committee, and all the experiments were carried out in accordance with the institutional guidelines on the care and use of experimental animals. A total of 50 µl from virus stock was injected for virus propagation. After 4 days of JEV injection, the mice were deeply anesthetized with chloroform and euthanized in aseptic condition in BSL-2 facility. The mice brains were harvested in aseptic condition and freezed immediately at − 70 °C and later homogenized in sterile phosphate buffered saline (PBS) and centrifuged at 13,000 rpm for 30 min at 4 °C. The supernatant was collected, aliquoted and stored at − 70 °C till further use. The virus titration was done on stable porcine kidney (PS) cell line by plaque assay as per standard procedure.

### To show JEV infection present with in the brain: mice inoculation, clinical sign/symptoms and sacrifice schedule

BALB/c mice were procured from the Indian Institute for Toxicology Research (IITR), Lucknow, India and housed at Sanjay Gandhi Post-Graduate Institute of Medical Sciences (SGPGIMS) Lucknow. Thirty (30) mice were randomly distributed in two groups. One is control group and another is virus infected group.

For infected group, 15 mice were inoculated with JEV (strain GP-78668A) of 3 × 10^6^ Plaque forming units (PFU) by intra-cerebral route (IC). Sterile phosphate buffered saline (PBS) was inoculated in rest 15 mice (age and sex matched control) and used as mock control group. Mice were monitored daily for sign, symptoms of encephalitis and survival. The mice were euthanized aseptically 0, 1, 3, 5 and 7 day post infection. At each time point, 3 mice each from both control and virus-infected groups were selected. Brain was excised aseptically and stored at − 80 °C for future study.

#### Body weight

Body weights of mice from both, the virus infected and control groups were assessed post JEV infection at 0, 1, 3, 5 and 7 days post infection.

#### Blood brain barrier integrity test for control and JEV infected mice

BBB integrity was evaluated with Evan’s blue dye exclusion test. Mice were grouped as described above i.e. control group and JEV infected group. Three mice each from control and JEV infected group (IC injection) at each time points i.e. 1, 3, 5 & 6 dpi were injected intravenously with 75 µl of 2% Evan’s blue (Sigma) in PBS. One hour later, mice were sacrificed and transcardially perfused with 10 ml of normal saline. Brains were then removed and photographed.

#### Plaque assay

In brief, PS cell monolayer was grown in 6-well tissue culture plates (Nunc, Denmark). Ten-fold serial dilutions of the propagated virus were made with 2% minimum essential medium (MEM). To the 75–80% confluent PS cell monolayer, 200 µl of each serial dilution was added and adsorbed by frequent shaking for 1 h at 37 °C with 5% CO_2_. Only MEM was used as a negative control. After adsorption, the cell monolayers were washed with PBS to remove the unadsorbed virus particles. To each well, 3 ml overlay media (a mixture of equal volumes of 1%agarose (kept at 45 °C) and 2X MEM (kept at 37 °C) was poured and allowed to solidify at room temperature (RT). The plates were then incubated for 4 days at 37 °C with 5% CO_2_. After 4 days of incubation, the cells were fixed by adding 10% formaldehyde for 2 h at RT. The overlay media were carefully removed with spatula and the cells were stained with 1% crystal violet. Plaques at different dilutions were counted manually and the dilution showing number of plaques in the range of 20–200 was selected for determination of virus titer using the following formula:

Once we had known virus titer by plaque assay we calculated the required multiplicity of infection (MOI) for known number of cells to get infected.

### Histology & immunohistochemistry (IHC)

Histopathology & IHC of brain tissues were carried out in the Department of Pathology, SGPGIMS, Lucknow, India. The mice were anaesthetized with ether and perfused with 150 ml PBS (0.1 M, pH 7.2). The brain were sliced and fixed in 10% buffered formalin for 3 days. After fixation the tissues were sectioned in 3–5 mm slices and processed overnight. Next day, the tissues were embedded in paraffin blocks. Five micron section were cut and dewaxed in xylene and graded alcohol (absolute 90%, 70%) and brought to water. One section from each block was cut and stained with hematoxylin–eosin (HE). Histopathological evaluation was done by the pathologist. The pathologist who was evaluating the sections was blinded for the treatment group.

For IHC, the sections were cut and lifted on polylysine coated slides and brought to water as above. Antigen retrievel was done in citrate buffer for half an hour at 98 °C & pH 6.0. Endogenous blocking was done by 3% H_2_O_2_ & washed in between with TRIS buffered saline. Primary antibody (clone NS1 for JEV protein detection at 1:25 dilution from abcam, USA) was applied for 2 h at room temperature and washed with TRIS buffered saline. Then, Secondary antibody (Universal Envision, DAKO) was applied for 30 min and then washed with TRIS buffered saline and then treated with chromogen (Diaminobenzidine). The slides were counterstained with hematoxylin and mounted with DPX.

### Microglial activation in response to JEV infection in mice brain

To investigate microglial activation in mice brain of JEV infected and control group, mRNA levels of ionized calcium-binding adapter molecule-1 (Iba-1, a specific microglial marker) were measured. Activated microglia contributes to the production of inflammatory cytokines that mediate direct or indirect neuron death.

### Establishment of JEV infection in microglial cell line BV2 and cell harvest at specific time points post-infection

BV2 cells were maintained in Dulbecco's modified Eagle's medium supplemented with 5% sodium bicarbonate (NaHCO_3_), 10% (FBS), 100 units/mL penicillin, and 100 μg/mL streptomycin at 37 °C with 5% CO_2_. BV2 cells were grown in six-well plates (Nunk Roskilde, Denmark). We trypsinized the cells and counted before growing in six-well plates. The BV2 cells were plated at the density of 3 × 10^6^ cells. Once the uniform monolayer of cells was grown, the medium of freshly grown BV2 cell line was removed and the cells were infected with JEV at a multiplicity of infection (MOI) of 5 and incubated at 37 °C in 5% CO_2_ for 1 h for virus adsorption. Post virus adsorption the BV2 cells were fed with fresh media and incubated at 37 °C in 5% CO_2_ for specific time intervals (0 h, 3 h, 6 h, 12 h and 24 h respectively). Mock infected controls were also used for each specific time points. For Mock-infected controls we used cell culture media (DMEM with 2% FBS) in place of virus. Cells were harvested immediately at specific time points from both the virus infected and mock-infected controls. For cell harvest, gentle scrapping was done using cell scraper and cells were collected aseptically and stored immediately at − 70 °C for 30 min. The harvested cells were taken out from − 70 °C and freezed- thawed for 3 cycles at − 70 °C and room temperature respectively. After freezed-thawed the cells were centrifuged at 1500 rpm and cell supernatant were collected in separate tubes and used for inflammatory mediator analysis and plaque assay for virus titer determination. The above steps were repeated for harvested cells collected at each specific time points post infection (tppi).

### Establishment of JEV infection in Neuronal cell line Neuro 2A and cell harvest at specific time points post-infection

The Neuro 2A cells were grown in six-well plates (Nunc). We trypsinized the cells and counted before growing in six-well plates. Once the uniform monolayer of cells was grown, the medium of freshly grown Neuro 2A cell line was removed and the cells were infected with JEV at a multiplicity of infection (MOI) of 5 and incubated at 37 °C for 1 h for virus adsorption. Post virus adsorption the Neuro 2A cells was fed with fresh media and incubated at 37 °C for specific time points. Mock infected controls were also used for each specific time points. For Mock-infected controls we used cell culture media (DMEM with 2% FBS) in place of virus. Cells were harvested immediately at specific time points from both the virus infected and mock-infected controls. For cell harvest, the cell scraper was used and cells were collected aseptically and stored immediately at − 70 °C till further use for inflammatory mediator analysis or plaque assays. For inflammatory mediator analysis or plaque assays the harvested cells were taken out from − 70 °C and freezed- thawed for 3 cycles at − 70 °C and room temperature respectively. After freezed-thawed the cells were centrifuged at 1500 rpm and cell supernatant were collected in separate tubes and used for inflammatory mediator analysis and plaque assay. The above steps were repeated for harvested cells collected at each specific time points post infection tppi.

### Quantification of JEV in BV2 and neuro 2A cell line at different time points post infection (tppi) by real time PCR

RNA was extracted using the QIAamp Viral RNA Mini Kit (QIAGEN) from culture supernatants obtained from control and treated BV2 cells and Neuro 2A cells according to the manufacturer’s instruction. To detect and quantify the JEV RNAs, commercially available kit (Genome diagnostic) was used according to the manufacturer’s instruction. Primers were specific for a 130 bp region of the JEV envelop gene. The probe was labeled with the reporter dye FAM at the 50 end and the quencher dye TAMRA at the 30 end. The reaction condition was 50 °C for 15 min, 95 °C for 10 min, 45 cycles of 94 °C for 10 s, 55 °C for 20 s, and 72 °C for15 s, using the ABI 7500 Real Time PCR System (Applied Biosystems, Foster City, CA, USA). Water (PCR grade) was used as a negative control.

### Primers used for the study

For quantitative Real time PCR (qRT-PCR), oligonucleotide primers for each specific TLRs, cytokine and chemokines were designed using free online primer design tool (http://bioinfo.ut.ee/primer3-0.4.0/primer3/) i.e. “Primer3 Input (version 0.4.0)”. The selected primers were subjected to an extensive search using BLAST tool (www.ncbi.nlm.nih.gov/blast) for species specificity. Following Primers were used for PCR amplification of target genes (Table 1).

**Table 1:** Primers sequences for expression study of TLRs, cytokines and chemokines

Gene name	Primer sequence (5’-3’)	Location	Gene bank ID
TLR-2	F-GCAAACGCTGTTCTGCTCAG	56–75	NM_011905
	R-AGGCGTCTCCCTCTATTGTATT	286–265	
TLR-4	F-AGGCACATGCTCTAGCACTAA	287–307	NM_021297
	R-AGGCTCCCCAGTTTAACTCTG	368–348	
TLR-9	F-ATGGTTCTCCGTCGAAGGACT	1–21	NM_031178
	R-GAGGCTTCAGCTCACAGGG	118–100	
IFN-γ	F-ATGAACGCTACACACTGCATC	1–21	NM_008337
	R-CCATCCTTTTGCCAGTTCCTC	182–162	
IL-6	F-CCAAGAGGTGAGTGCTTCCC	462–481	NM_031168
	R-CTGTTGTTCAGACTCTCTCCCT	579–558	
IL-17	F-TCT GAT GCT GTT GCT GCT G	87–105	NM_010552.3
	R-ACG GTT AGA GGT AGT CTG AGG	254–267	
IL-21	F-GGACCCTTGTCTGTCTGGTAG	8–28	NM_021782
	R-TGTGGAGCTGATAGAAGTTCAGG	173–151	
Foxp3 + regulatory T cells	F- GGC CCT TCT CCA GGA CAG A	551–570	NM_054039.2
	R-GCT GAT CAT GGC TGG GTT GT	642–662	
RANTES(CCL-5)	F-GCTGCTTTGCCTACCTCTCC	98–117	NM_013653
	R-TCGAGTGACAAACACGACTGC	201–181	
MCP-1(CCL-2)	F-TTAAAAACCTGGATCGGAACCAA	260–282	NM_01133 3
	R-GCATTAGCTTCAGATTTACGGGT	380–358	
β-actin	F-TGG AAT CCT GTG GCA TCC ATG AAA C	885–909	NM_007393.3
	R-TAA AAC GCA GCT CAG TAA CAG TCC G	1209–1233	

### Quantification of TLRs, cytokines and chemokines levels in BV2 and Neuro 2A cell line at different time points post infection (tppi) by real time PCR

The TLRs, cytokines and chemokines levels in culture supernatants obtained from control and treated BV2 and Neuro2Acells were quantified using quantitative TaqMan -based real-time qRT-PCR. Total RNAs from culture supernatant were extracted as per manufacturer’s instructions. RNA was quantified by measuring the optical density at 260 nm (OD_260_) using a Nanodrop-1000 spectrophotometer (Thermo Scientific) and the purity was assessed by determining the OD_260_/OD_280_ ratio, which was between 1.9 and 2.One µg of extracted RNA was then used in real-time qRT-PCR. Following reverse transcription of total RNAs using a High-Capacity cDNA Reverse Transcription Kit (Applied Biosystems, Foster City, CA, USA), a reaction mixture containing 1 µl of template cDNA, 10 µl of 2 × Premix x Taq, and 600 nM primers in a final volume of 20 µl was prepared. No template control (NTC) reactions were also performed along with the PCR to identify PCR contamination. The conditions used for qRT-PCR are as follows: 95 °C for 2 min (hold), 94 °C for 15 s, 60 °C for 15 s, and 72 °C for 1 min (40 cycles). All samples were run in triplicate. The dissociation curves were generated to check for the specificity of primer annealing to the template. The levels of cytokines, chemokines and TLRs mRNAs were normalized to the levels of the rat housekeeping gene β-actin of the same sample. Relative changes of gene expression were calculated by the 2^−ΔΔCt^ formula, and the data are represented as percentage change of the control group.

### Statistical analysis

Data are represented as mean ± standard deviation (SD). Statistical analyses for continuous data were performed with Prism6 for Windows software (Prism Graph-Pad Software Inc.). *p* < 0.05 was considered significant. Graphs were produced, and statistical analyses were performed using GraphPad Prism.

## Results

### Clinical findings of in vivo infection (mice survival and clinical signs and symptoms)

**Fig. 1:**
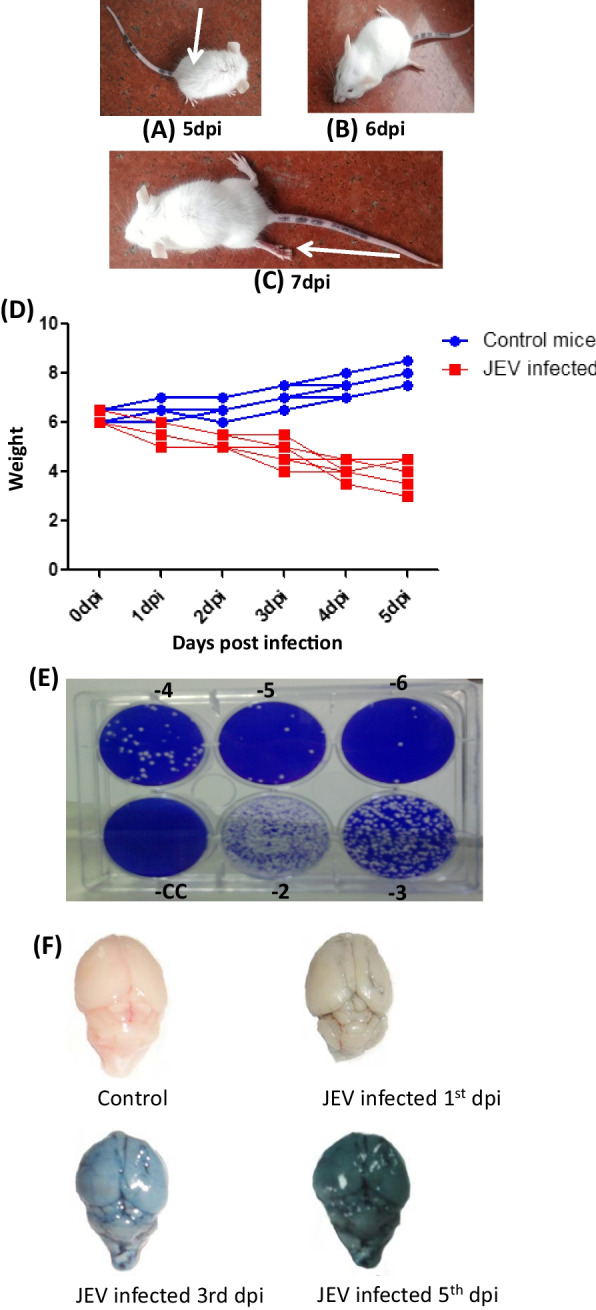
**A**–**C** Virus propagation in mice brain: Mice were inoculated intracerebral (IC) with GP-78 strain of Japanese encephalitis virus (**A**) Hunching of the back (indicated by arrow), and slight hind limb disability was observed on 5 dpi. **B**, **C** The typical symptoms of encephalitis characterized by total hind limb paralysis (indicated by arrow), tremors, and convulsions with altered consciousness became evident on 6 and 7 dpi. The mice were almost moribund when they were sacrificed. **D** Balb/c mice were infected with JEV. PBS inoculated mice were used as mock control. Body weight of mice from both the groups was measured at different day post infection. The drop in body weight of JEV-infected mice was noticed compared to mock controls. **E** Six well tissue culture plates demonstrating the virus titration from the brain of intracerebrally inoculated mice. **F** Blood brain barrier damage of mice infected with JEV (IC challenge) by Evan’s blue dye exclusion test

All the mice in the mock infected controls were healthy with no clinical sign and symptoms at any time point. In the virus infected mice, 100% mortality was observed after 7 dpi. Thus, we have choosen time point upto 6dpi in our study group. In the virus infected group, hunching of the back and slight hind limb disability was observed on 4 dpi. The typical symptoms of encephalitis characterized by total hind limb paralysis, tremors, and convulsions with altered consciousness became evident on 5 dpi and 6 dpi (Fig. 1A–C). Mice were almost moribund when they were sacrificed. The mice brain were harvested in aseptic condition at BSL-3 facility of SGPGIMS, Lucknow and stored at − 80 °C until further use.

#### Body weight

Drop in body weight of JEV-infected mice was noticed at each time points compared to mock control (Fig. 1D).

### Virus growth assay

To study the virus multiplication in the brain of virus infected mice, at each time points of sacrifice, the virus titer was determined by Plaque assay. A significant increase from 9.9 × 10^6^ PFU/g at 3 dpi to 1.3 × 10^8^ PFU/g at 5 dpi was noted. This increased further to 2.0 × 10^9^ PFU/g in the brain of virus infected mice at 6 dpi (Fig. 1E).

### JEV infection damages the blood brain barrier (BBB) in mice

Evan’s blue is a cationic dye that binds to serum albumin to form a dye- protein complex that cannot pass through the intact BBB. Due to this, it is used as a standard molecule to test the permeability of the BBB. Intravenous injection of Evan’s blue results in the formation of a conjugate with serum albumin. Inclusion of the blue colored dye in the brain indicated the passage of the dye protein conjugate into the brain that signifies increased permeability or damage of BBB. Mice from control group and JEV infected group was taken to test the BBB permeability (Fig. 1F). Compared to mock-infected control, the leakage of Evan’s blue dye in the JEV infected mice brain started at 3 dpi which was more prominent at 6 dpi. This clearly indicates the compromised BBB integrity in virus infected mice brain with respect to control.

### Histopathology

#### Neuropathological changes

**Fig. 2:**
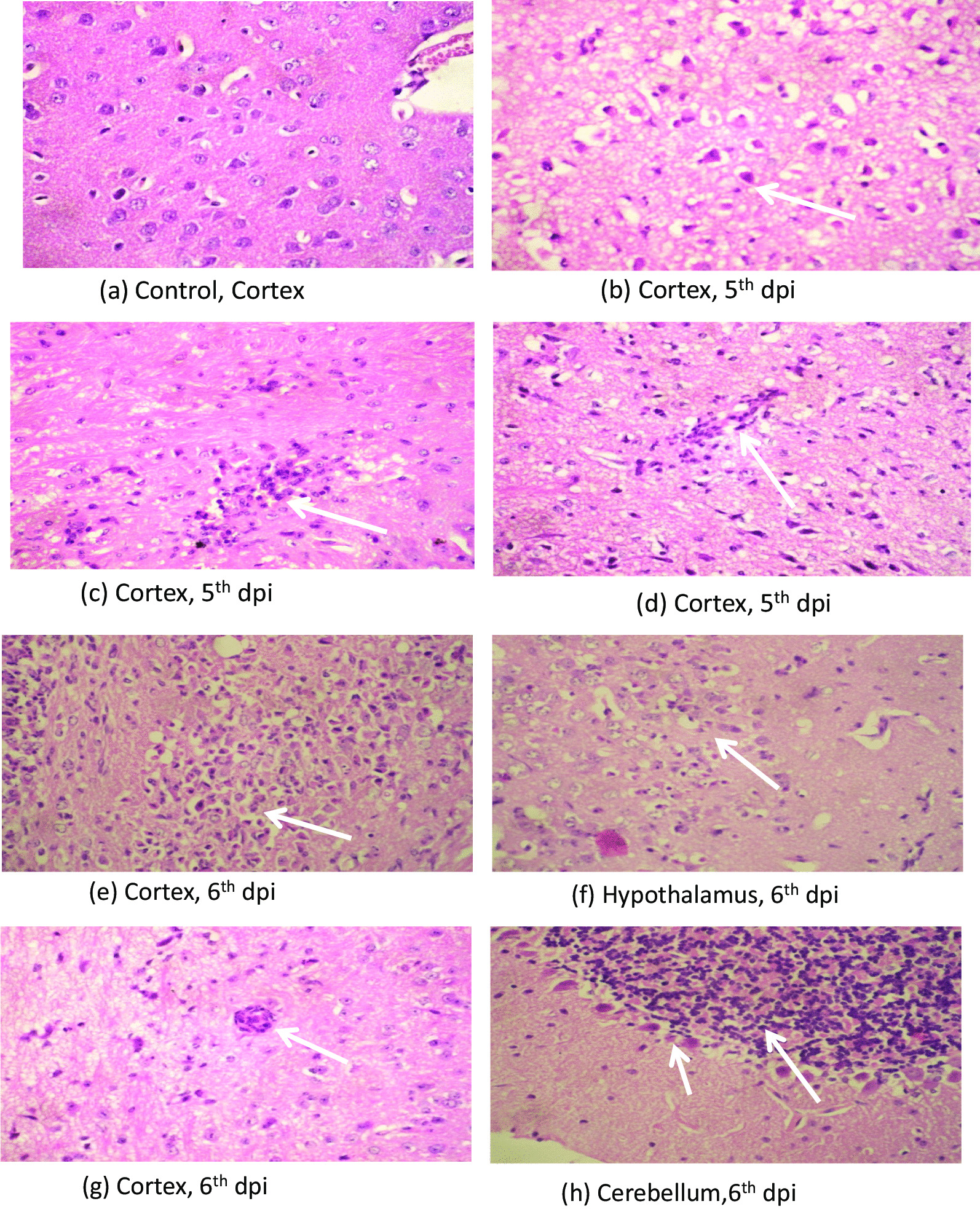
Photomicrograph of H & E staining showing (**a**) normal appearing neurons with round nuclei, vascular chromatin, prominent nuclei and moderate cytoplasm in cortex of control mice brain, (**b**) neuronal shrinkage with shrunken nuclei and hypereosinophilic cytoplasm, (**c**) inflammatory cell infiltrate with in the brain parenchyma causing neuronal damage, (**d**) perivascular inflammatory cells and neuronal damage at 5th dpi (Magnification at X40). Photomicrograph of H & E staining showing intense (**e**) inflammatory infiltration causing necrosis of brain parenchyma, (**f**) inflammatory infiltrate and neuronal damage, (**g**) perivascular inflammation in the cortex, (**h**) Cerebellum showing neuronal shrinkage & hypereosinophilia of the purkinjee cells at 6th dpi (Magnification at X40)

Section from mouse brain stained with HE showed neuronal damage in the form of neuronal shrinkage and hypereosinophilia. There was edema, spongy degeneration and infiltration by neutrophil and lymphocytes within the brain parenchyma around neurons more in the thalamic and hypothalamic area followed by cerebral cortex. Perivascular inflammatory infiltrate was seen. Cerebellum showed only mild neuronal damage displaying shrinkage and hypereosinophilia (Fig. 2a–h). These changes in the brain parenchyma were appreciable at 5th dpi and were more intense on 6th dpi.

#### Immunohistochemistry (IHC)

**Fig. 3:**
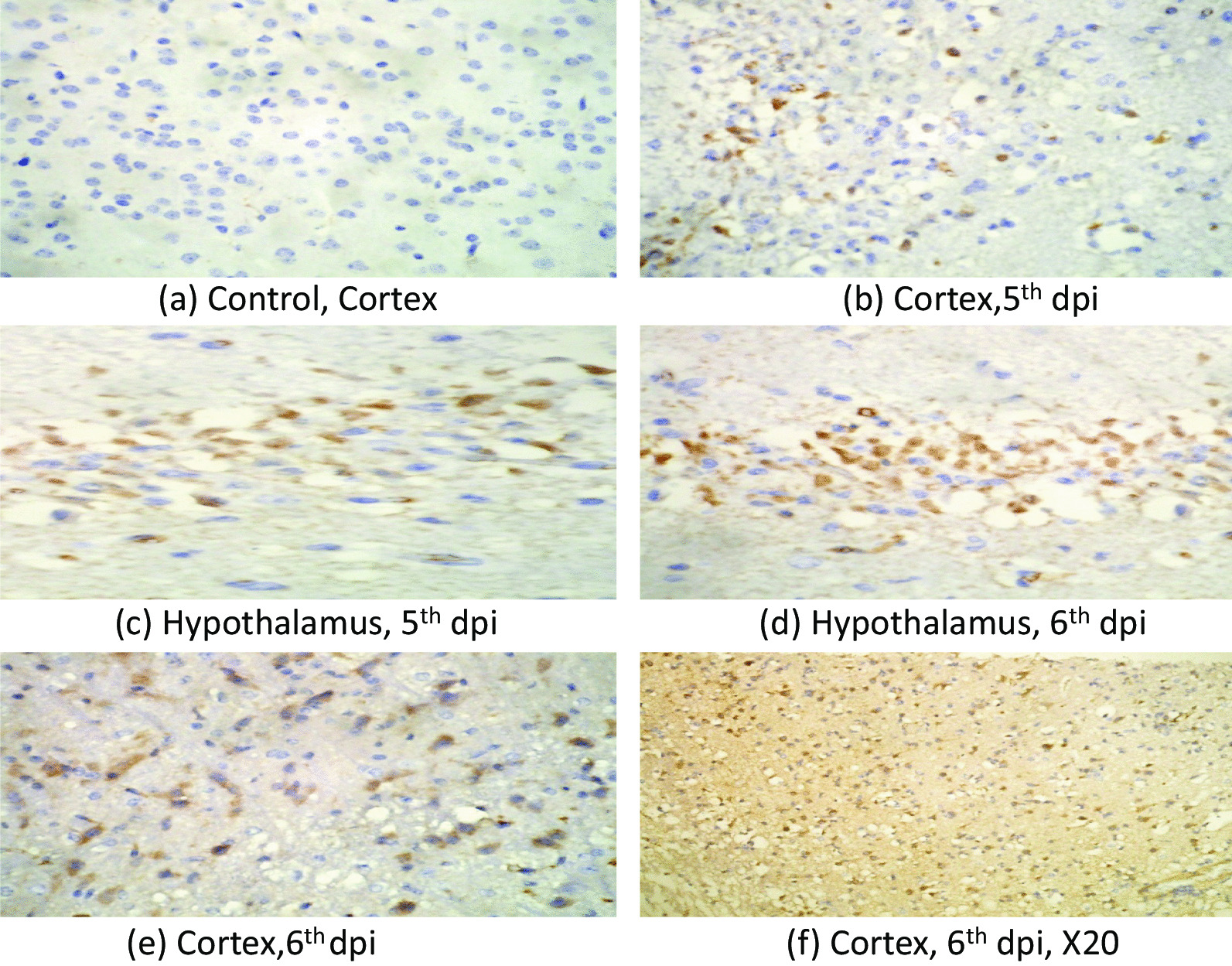
Photomicrograph showing presence of JEV antigen in cortex and hypothalamus region of BALB/c mouse brain infected with 3X10^6^ pfu /ml of JEV(GP-78) at 5th and 6th dpi

IHC was performed to look for the presence JEV. Our IHC results showed JEV infected neurons in the hypothalamus, thalamus and cortex. Cerebellum neurons did not show presence of JEV NS1 antigen. The antigen was first visible at 5th day post infection and more pronounced on the 6th day post infection (Fig. 3a–f).

**Fig. 4:**
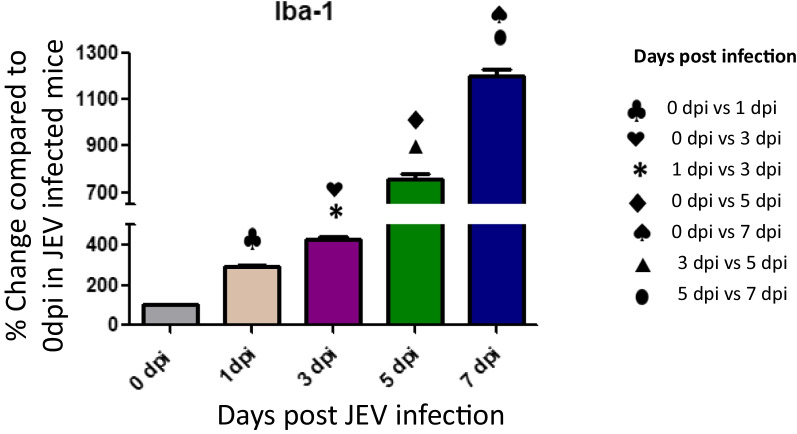
Expression of Iba-1 in JEV infected mice at different (0,1,3,5 and 7 dpi) dpi. Balb/c mice were infected with JEV. mRNA expression was analyzed at different dpi by qPCR. Bar Graph represents the quantification of Iba-1 normalized to β-actin. Values are means ± SD of 3 animals at each dpi

**Fig. 5:**
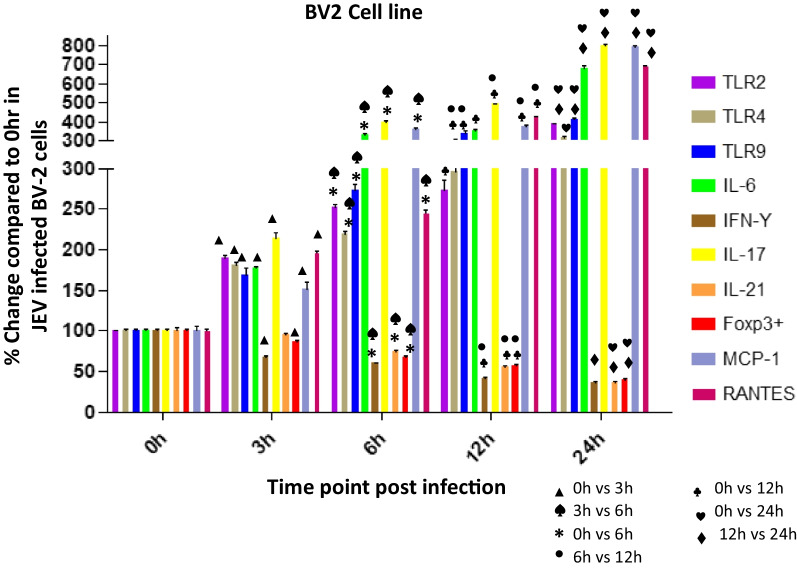
Expression of TLRs (2, 4, 9), IL-6, IFN-ϒ, IL-17, IL-21, Foxp3 + , MCP-1, RANTES in Japanese encephalitis virus (JEV) infected BV-2 cells. BV-2 was infected with JEV for 0, 3, 6, 12, or 24 h. mRNA expression was analyzed by qPCR. Bar Graph represents the quantification of (2, 4, 9), IL-6, IFN-ϒ, IL-17, IL-21, Foxp3 + , MCP-1, RANTES normalized to β-actin. Data represent mean ± SEM from three independent experiments

**Fig. 6:**
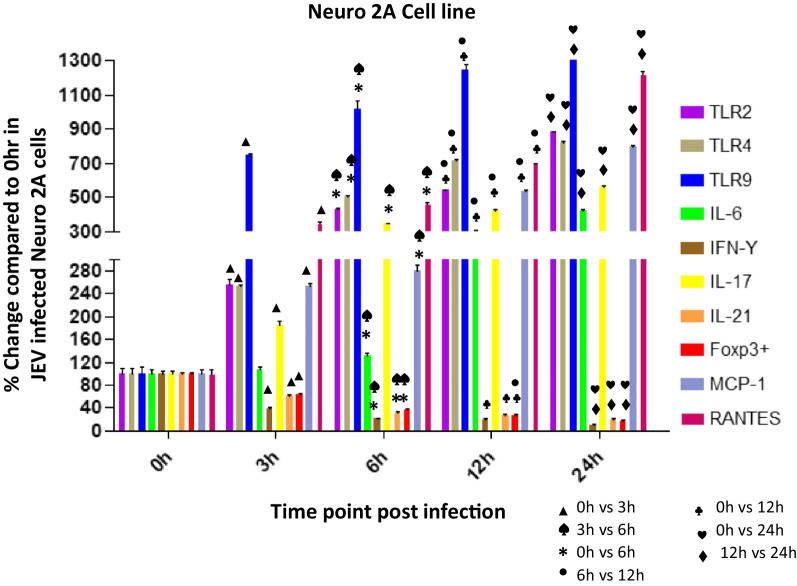
Expression of TLRs (2, 4, 9), IL-6, IFN-ϒ, IL-17, IL-21, Foxp3 + , MCP-1, RANTES in Japanese encephalitis virus (JEV) infected Neuro 2A cells. Neuro 2A was infected with JEV for 0, 3, 6, 12, or 24 h. mRNA expression was analyzed by qPCR. Bar Graph represents the quantification of (2, 4, 9), IL-6, IFN-ϒ, IL-17, IL-21, Foxp3 + , MCP-1, RANTES normalized to β-actin. Data represent mean ± SEM from three independent experiments

Upregulated microglia activation was noticed in JEV infected mice brain when compared to mock infected controls (Fig. 4). The result clearly indicates that JEV infection induces inflammation in the brain by activation of microglial cells. Activation of microglial cells might play a significant role in inducing neuronal cell death by stimulating the production of proinflammatory mediators. Several authors reported that the levels of various proinflammatory mediators, such as IL-6, IL-1β, TNF-α, and MCP-1, were significantly elevated in microglial cells following JEV infection (Figs. 5, 6).

#### JEV infects both BV2 and neuro 2A cell lines

Microglia is the resident mononuclear immune cells of the CNS and play important roles in the maintenance of homeostasis and neuroinflammation. We measured viral loads (GP78 mRNA levels) in JEV-infected BV2 cells and Neuro 2A cells. BV2 and Neuro 2A cells were seeded in 6-well plates a day before infection. The cells were infected at a multiplicity of infection (MOI) of 5 with plaque-determined titers of JEV. Cells were observed daily for change in morphology if any post-JEV infection. A change in cell morphology was observed after virus infection starting at 3 h post infection. At later time points i.e. 12 h and 24 h (post infection) due to progressive JEV infection (JEV multiplication) the CPE (cytopathic effect) in cells were more evident.

**Fig. 7:**
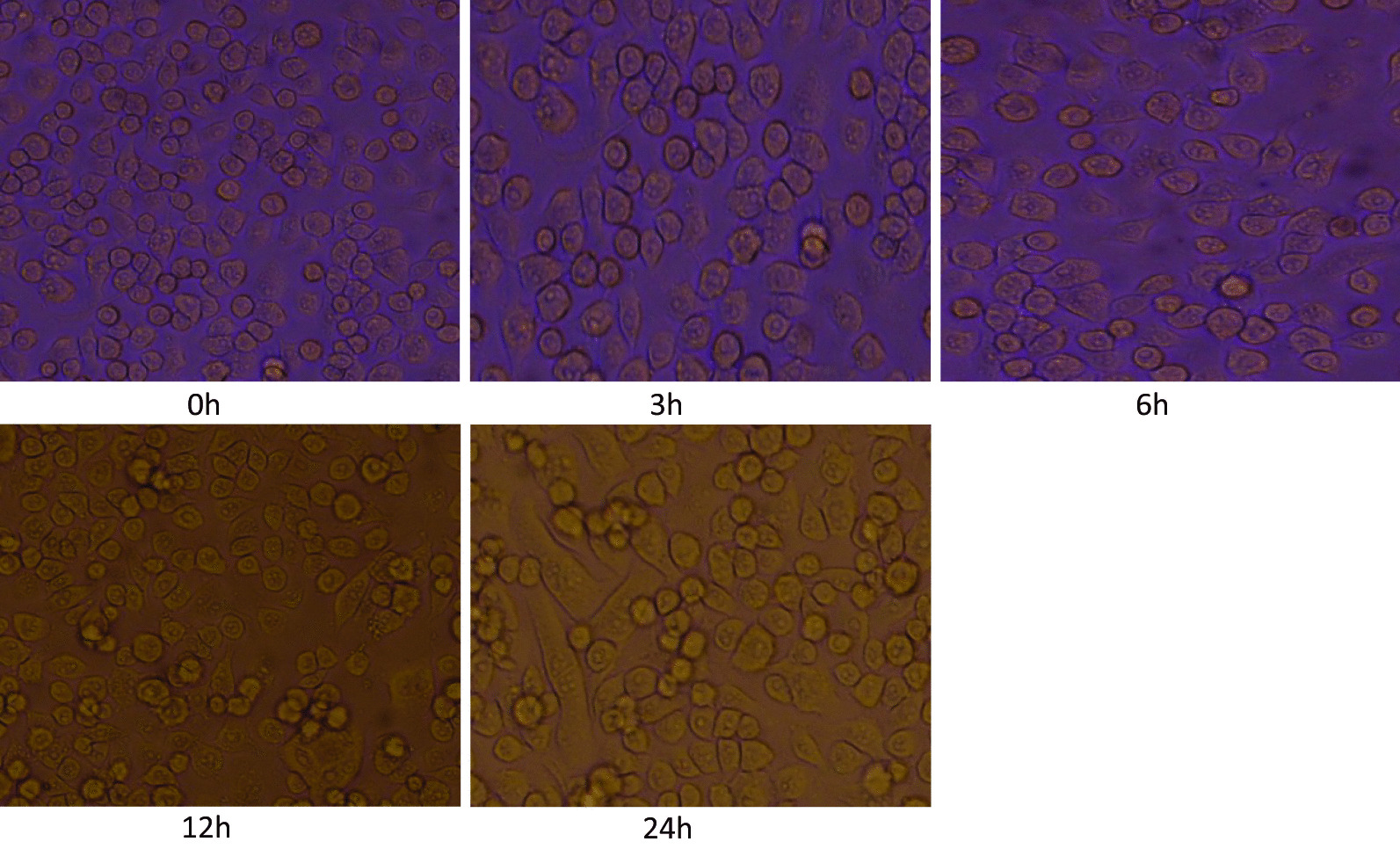
BV2 cells at different time post infection with JEV (0 h, 3 h, 6 h, 12 h, 24 h). BV2 cells were seeded in 6-well plates a day before infection. The cells were infected at a multiplicity of infection (MOI) of 5 with plaque-determined titers of JEV. Cells were observed daily for change in morphology if any post-JEV infection

**Fig. 8:**
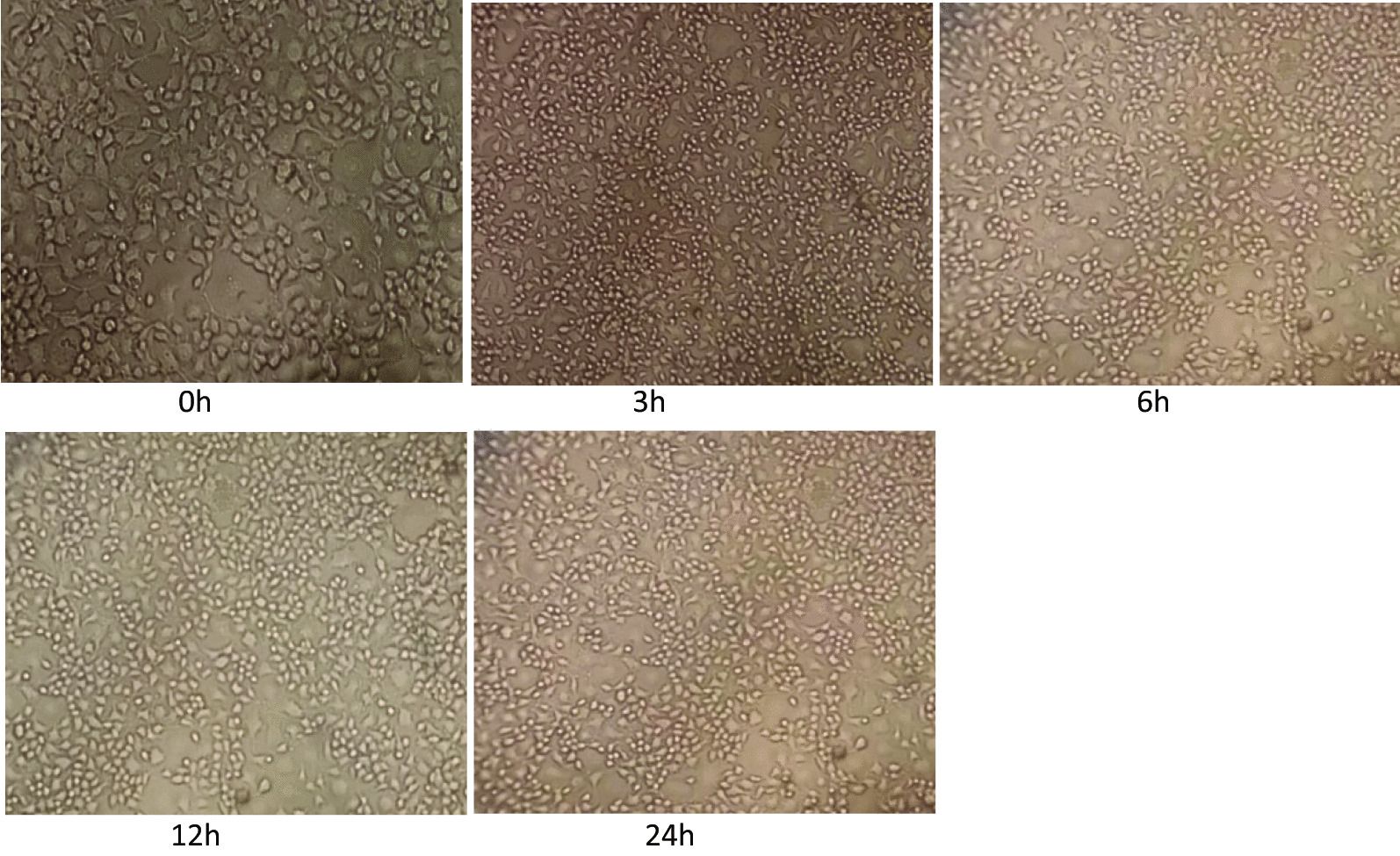
Neuro2A cells at different time post infection with JEV (0 h, 3 h, 6 h, 12 h, 24 h). Neuro 2A cells were seeded in 6-well plates a day before infection. The cells were infected at a multiplicity of infection (MOI) of 5 with plaque-determined titers of JEV. Cells were observed daily for change in morphology if any post-JEV infection

Our result showed that JEV infect both BV2 cells and Neuro2A cells as observed by cytopathic effect (Figs. 7 and 8).

### Expression levels of TLRs, cytokines, and chemokines post JEV infection in BV2 cell line at various time points

To address the question, whether the levels of TLRs and associated cytokines/ chemokines get deregulated post JEV infection, we infected the freshly grown microglial cells and neuronal cells with JE virus at the MOI of 5 and incubated at 37 °C with 5%CO_2_for various time intervals i.e. at 3 h, 6 h, 12 h, 24 h respectively. The culture supernatants from JEV infected microglial cells and mock infected controls were collected at specified time points mentioned above. The cells were also collected immediately after JEV infection and it was assigned as 0 h.

Upon JEV infection at a MOI of 5, the cytopathic effect (CPE) in cells was observed starting at 3 h and at 12 h and 24 hpi the CPE was more evident.

### Upregulated mRNA levels of TLR-2, TLR-4 and TLR-9 in BV2 cells post JEV infection

#### TLR2

The level of TLR2 was significantly increased with increasing time points post infection. At 3 h post infection (pi) the TLR2 level was increased to 191% (*p* < 0.0001) which increased significantly at 6 h (253%, *p* < 0.0001), 12 h (273%,* p* < 0.0001) and reached to the level 386% (*p* < 0.0001) at 24 h post infection (Fig. 5) when compared to the level expressed at 0 h post JEV infection. A significant 133%, *p* < 0.0001 increase in TLR 2 level at 6 h was noticed compared to 3 h post JEV infection. But the increase at 12 h was not significant compared to 6 h post infection. At 24 h the level was significantly increased by 141%, *p* < 0.0001 compared to 12 h post infection. Overall we noticed an increase in TLR2 level with increasing time points post infection.

### TLR4

Like TLR2, the level of TLR4 was significantly increased with increasing time points post infection. At 3 h post infection the TLR4 level was 180% which increased significantly at 6 h (220%, *p* < 0.0001), 12 h (297%, *p* < 0.0001) and reached to the level 312 (*p* < 0.01) at 24 h post infection (Fig. 5) when compared to the level expressed at 0 h post JEV infection. A significant 122%,* p* < 0.0001 increase in TLR 4 level at 6 h was noticed compared to 3 h post JEV infection. The level increased to 135% *p* < 0.001 at 12 h compared to 6 h post infection. At 24 h the level was not increased significantly compared to 12 h post infection. Overall we noticed an increase in TLR4 level with increasing time points post infection.

### TLR9

The level of TLR9 was 168%, *p* < 0.0001 at 3 h post JEV infection which dramatically increased with increasing time points post infection and reached to the level 414%, *p* < 0.0001 at 24 h post infection (Fig. 5) when compared to the level expressed at 0 h post JEV infection. A significant 162%,* p* < 0.0001 increase in TLR 9 level at 6 h was noticed compared to 3 h post JEV infection. The TLR9 level was increased significantly by 123%, *p* < 0.01 at 12 h compared to 6 h post infection. Again, at 24 h the level was increased significantly by 123%, *p* < 0.0001 compared to 12 h post infection. Overall we noticed a dramatic increase in TLR9 level with increasing time points post infection.

### Upregulated mRNA levels of IL-6 and IL-17 in BV2 cells post JEV infection

#### IL-6

We noticed a drastic increase in IL6 level with increasing time points post infection. At 3 h pi the IL6 level was 177%, *p* < 0.0001 which increased significantly to the level 332%, *p* < 0.0001at 6 h and 353%, *p* < 0.0001 at 12 h and the level shoot-up to 682%, *p* < 0.0001 at 24 h post infection (Fig. 5) when compared to the level expressed at 0 h post JEV infection. A significant 188%, *p* < 0.0001 increase in IL6 level at 6 h was noticed compared to 3 h post JEV infection. The IL-6 level was almost same at 12 h compared to 6 h post infection. At 24 h the IL-6 level was increased significantly by 193%, *p* < 0.0001 compared to 12 h post infection. Overall we noticed a drastic increase in IL6 level at later time points post infection compared to initial time points.

### IL-17

Like IL-6 levels, we noticed a drastic increase in IL-17 level with increasing time points post infection. At 3 h pi the IL-17 level was 214%, *p* < 0.0001 which increased significantly to the level 400%, *p* < 0.0001 at 6 h and 493%, *p* < 0.0001 at 12 h and the level shoot-up to 800%, *p* < 0.0001 at 24 h post infection (Fig. 5) when compared to the level expressed at 0 h post JEV infection. A significant 187%,* p* < 0.0001 increase in IL-17 level at 6 h was noticed compared to 3 h post JEV infection. The IL-17 level was increased significantly by 123%, *p* < 0.0001 at 12 h compared to 6 h post infection. At 24 h the IL-17 level was increased significantly by 163%, *p* < 0.0001 compared to 12 h post infection. Overall we noticed an exaggerated increase in IL-17 level at later time points post infection compared to initial time points.

### ***Downregulated mRNA levels of IL-21, FoxP3***^+^*** and IFN-ϒ in BV2 cells post JEV infection***

#### IL-21

The level of IL-21 was decreased with increasing time points post infection. At 3 h post infection the IL-21 level was 95% which decreased significantly at 6 h (74%, *p* < 0.0038), 12 h (55%, *p* < 0.0005) and finally decreased to the level 37% (*p* < 0.0001) at 24 h post infection (Fig. 5) when compared to the level expressed at 0 h post JEV infection. A significant 77%, *p* < 0.0034 decrease in IL-21 level at 6 h was noticed compared to 3 h post JEV infection. The IL-21 level was further decreased significantly to 74%, *p* < 0.0042 at 12 h compared to 6 h post infection. At 24 h the level was further decreased by 67%, *p* < 0.0001 compared to 12 h post infection. Overall we noticed a significant decrease in IL-21 level at later time points compared to initial time points post infection.

#### ***FoxP3***^+^

The level of FoxP3^+^ was decreased significantly with increasing time points post infection. At 3 h post infection the FoxP3^+^level was 87%, *p* < 0.0086 which decreased significantly at 6 h (67%, *p* < 0.0003), 12 h (57%, *p* < 0.0001) and finally decreased to the level 40% (*p* < 0.0001) at 24 h post infection (Fig. 5) when compared to the level expressed at 0 h post JEV infection. A significant 77%, *p* < 0.002 decrease in FoxP3^+^level at 6 h was noticed compared to 3 h post JEV infection. The FoxP3^+^level was further decreased significantly to 85%, *p* < 0.02 at 12 h compared to 6 h post infection. At 24 h the level was further decreased by 71%, *p* < 0.002 compared to 12 h post infection. Overall we noticed a significant decrease in FoxP3^+^ level at later time points compared to initial time points post infection.

#### IFN-ϒ

The level of IFN-ϒ was decreased significantly with increasing time points post infection. At 3 h post infection the IFN-ϒ level was decreased to 68%, *p* < 0.0086 which decreased significantly at 6 h (60%, *p* < 0.0003), 12 h (42%, *p* < 0.0001) and finally decreased to the level 37% (*p* < 0.0001) at 24 h post infection (Fig. 5) when compared to the level expressed at 0 h post JEV infection. A significant 88%, *p* < 0.002 decrease in IFN-ϒ level at 6 h was noticed compared to 3 h post JEV infection. The IFN-ϒ level was further decreased significantly to 71%, *p* < 0.02 at 12 h compared to 6 h post infection. At 24 h the level was further decreased by 86%, *p* < 0.002 compared to 12 h post infection. Overall we noticed a significant decrease in IFN-ϒ level at later time points compared to initial time points post infection.

### Upregulated mRNA levels of chemokines MCP-1 and RANTES (regulated upon activation, normal T-cell expressed and secreted)in BV2 cells post JEV infection

#### MCP-1

The level of MCP-1 was significantly increased with increasing time points post infection. At 3 h post infection (pi) the MCP-1level was increased to 152% (p = 0.0073) which increased significantly at 6 h (363%, *p* < 0.0001), 12 h (379%, *p* < 0.0001) and reached to the level 795% (*p* < 0.0001) at 24 h post infection (Fig. 5) when compared to the level expressed at 0 h post JEV infection. A significant 240%, *p* < 0.0001 increase in MCP-1 level at 6 h was noticed compared to 3 h post JEV infection. But the increase at 12 h was not significant compared to 6 h post infection. At 24 h the level was significantly increased by 210%, *p* < 0.0001 compared to 12 h post infection. Overall we noticed an increase in MCP-1 level with increasing time points post infection.

### RANTES

The level of RANTES was significantly increased with increasing time points post infection. At 3 h post infection (pi) the RANTES level was increased to 197% (*p* < 0.0001) which increased significantly at 6 h (246%, *p* < 0.0001), 12 h (423%, *p* < 0.0001) and reached to the level 692% (*p* < 0.0001) at 24 h post infection (Fig. 5) when compared to the level expressed at 0 h post JEV infection. A significant 125%, *p* < 0.0001 increase in RANTES level at 6 h was noticed compared to 3 h post JEV infection. The level increase significantly to 172%, *p* < 0.0001 at 12 h compared to 6 h post infection. At 24 h the level was significantly increased by 164%, *p* < 0.0001 compared to 12 h post infection. Overall we noticed an increase in RANTES level with increasing time points post infection.

### Expression levels of TLRs, cytokines and chemokines post JEV infection in Neuro 2A cell line at various time points

To address the question, whether the levels of TLRs and associated cytokines/ chemokines get deregulated post JEV infection, we infected the freshly grown neuronal cells and neuronal cells with JE virus at the MOI of 5 and incubated at 37 °C with 5% CO_2_ for various time intervals i.e. at 3 h, 6 h, 12 h, 24 h respectively. The culture supernatants from JEV infected cells microglial cells and mock infected controls were collected at specified time points mentioned above. The cells were also collected immediately after JEV infection and it was assigned as 0 h.

Upon JEV infection at a MOI of 5, the cytopathic effect (CPE) in cells was observed starting at 3 h and at 12 h and 24 hpi the CPE was more evident.

### Upregulated mRNA levels ofTLR-2, TLR-4 and TLR-9 in Neuro 2A cells post JEV infection

#### TLR2

The level of TLR2 was significantly increased with increasing time points post infection. At 3 h post infection (pi) the TLR2 level was increased to 257% (*p* < 0.0001) which increased significantly at 6 h (432%, *p* < 0.0001), 12 h (543%, *p* < 0.0001) and increased to the level by 882% (*p* < 0.0001) at 24 h post infection (Fig. 6) when compared to the level expressed at 0 h post JEV infection. A significant 168%,* p* < 0.0001 increase in TLR 2 level at 6 h was noticed compared to 3 h post JEV infection. The increase at 12 h was 126%,* p* < 0.0001 compared to 6 h post infection. At 24 h the level was significantly increased by 163%, *p* < 0.0001 compared to 12 h post infection. Overall we noticed an increase in TLR2 level with increasing time points post infection.

#### TLR4

Like TLR2, the level of TLR4 was significantly increased with increasing time points post infection. At 3 h post infection the TLR4 level was increased to 253% which increased significantly at 6 h (506%, *p* < 0.0001), 12 h (716%, *p* < 0.0001) and reached to the level 823% (*p* < 0.0001) at 24 h post infection (Fig. 6) when compared to the level expressed at 0 h post JEV infection. A significant 200%,* p* < 0.0001 increase in TLR 4 level at 6 h was noticed compared to 3 h post JEV infection. The level increased to 142% *p* < 0.0001at 12 h compared to 6 h post infection. At 24 h the level was increased significantly to 114%,* p* < 0.0001 compared to 12 h post infection. Overall we noticed an increase in TLR4 level with increasing time points post infection.

#### TLR9

The level of TLR9 was increased to 747%, *p* < 0.0001 at 3 h post JEV infection which dramatically increased with increasing time points post infection compared. At 6 h the level was increased by 1013%, *p* < 0.0001, 12 h (1247%, *p* < 0.0001) which further increased by the level 1763% (*p* < 0.0001) at 24 h post infection (Fig. 6) when compared to the level expressed at 0 h post JEV infection. A significant 136%,* p* < 0.001 increase in TLR 4 level at 6 h was noticed compared to 3 h post JEV infection. The level increased to 123% *p* < 0.01at 12 h compared to 6 h post infection. At 24 h the level was increased significantly to 141%, *p* < 0.0001 compared to 12 h post infection. Overall we noticed a dramatic increase in TLR9 level with increasing time points post infection. The cytopathic effect was observed in Neuro2A cells at 12 h and 24 h post infection.

### Upregulated mRNA levels of IL-6 and IL-17 in Neuro 2A cells post JEV infection

#### IL-6

We noticed an increase in IL6 level later time points post infection. At 3 h pi the IL6 level was almost similar to the level at 0 h post infection. The level increased significantly by 301%, *p* < 0.0001 at 12 h and the level increased to 426%, *p* < 0.0001 at 24 h post infection (Fig. 6) when compared to the level expressed at 0 h post JEV infection. A significant 124%,* p* < 0.0238 increase in IL6 level at 6 h was noticed compared to 3 h post JEV infection. The IL-6 level was increased by 228%, *p* < 0.0001 at 12 h compared to 6 h post infection. At 24 h the IL-6 level was increased significantly by 142%, *p* < 0.0002 compared to 12 h post infection. Overall we noticed a major increase in IL6 level at later time points post infection compared to initial time points.

#### IL-17

Like IL-6 levels, we noticed an Upregulated expression of IL-17 level with increasing time points post infection. At 3 h pi the IL-17 level was 185%, *p* < 0.0006 which increased significantly to the level 346%, *p* < 0.0001 at 6 h and 426%, *p* < 0.0001 at 12 h and the level shoot-up to 562%, *p* < 0.0001 at 24 h post infection (Fig. 6) when compared to the level expressed at 0 h post JEV infection. A significant 187%, *p* < 0.0013 increase in IL-17 level at 6 h was noticed compared to 3 h post JEV infection. The IL-17 level was increased significantly by 123%, *p* < 0.0001 at 12 h compared to 6 h post infection. At 24 h the IL-17 level was increased significantly by 132%, *p* < 0.005 compared to 12 h post infection. Overall we noticed an exaggerated increase in IL-17level at later time points post infection compared to initial time points.

### Downregulated mRNA levels of IL-21, FoxP3^+^ and IFN-ϒ in Neuro2A cells post JEV infection

#### IL-21

The level of IL-21 was decreased with increasing time points post infection. At 3 h post infection the IL-21 level was 60% which decreased significantly at 6 h (30%, *p* < 0.0001), 12 h (27%, *p* < 0.0001) and finally decreased to the level 20% (*p* < 0.0001) at 24 h post infection (Fig. 6) when compared to the level expressed at 0 h post JEV infection. A significant 50%, *p* < 0.0013 decrease in IL-21 level at 6 h was noticed compared to 3 h post JEV infection. Overall we noticed a decrease in IL-21 level at later time points compared to initial time points post infection.

#### ***FoxP3***^+^

The level of FoxP3^+^was decreased significantly with increasing time points post infection. At 3 h post infection the FoxP3^+^ level was 63%, p = 0.0009 which decreased significantly at 6 h (36%, *p* < 0.0001), 12 h (27%, *p* < 0.0001) and finally decreased to the level 16% (*p* < 0.0001) at 24 h post infection (Fig) when compared to the level expressed at 0 h post JEV infection. A significant more than 40%, p = 0.0021 decrease in FoxP3^+^level at 6 h was noticed compared to 3 h post JEV infection. The FoxP3^+^ level was further decreased significantly to 36%, *p* < 0.04 at 12 h compared to 6 h post infection. At 24 h the level was further decreased by 39%, *p* < 0.02 compared to 12 h post infection. Overall we noticed a significant decrease in FoxP3^+^ level at later time points compared to initial time points post infection.

#### IFN-ϒ

The level of IFN-ϒ was decreased significantly with increasing time points post infection. At 3 h post infection the IFN-ϒ level was decreased to 38%, p = 0.0002 which decreased significantly at 6 h (22%, *p* < 0.0001), 12 h (%20, *p* < 0.0001) and finally decreased to the level 10% (*p* < 0.0001) at 24 h post infection (Fig. 6) when compared to the level expressed at 0 h post JEV infection. A significant approx 40%, p = 0.0058 decrease in IFN-ϒ level at 6 h was noticed compared to 3 h post JEV infection. The IFN-ϒ level was further decreased significantly to 51%, p = 0.02 at 24 h compared to 12 h post infection. Overall we noticed a significant decrease in IFN-ϒ level at later time points compared to initial time points post infection.

### Upregulated expression of chemokines MCP-1 and RANTES in Neuro 2A cells post JEV infection

#### MCP-1

The level of MCP-1 was significantly increased with increasing time points post infection. At 3 h post infection (pi) the MCP-1 level was increased to 253% (*p* < 0.0001) which increased significantly at 6 h (281%, *p* < 0.0001), 12 h (535%, *p* < 0.0001) and reached to the level 794% (*p* < 0.0001) at 24 h post infection (Fig. 6) when compared to the level expressed at 0 h post JEV infection. The MCP-1 level at 6 h was near to the level expressed at 3 h post JEV infection. The level was increased by 191%, *p* < 0.0001 at 12 h compared to 6 h post infection. At 24 h the level was significantly increased by 149%, *p* < 0.0001 compared to 12 h post infection. Overall we noticed an increase in MCP-1 level with increasing time points post infection. The cytopathic effect was observed in Neuro2A cells at 12 h and 24 h post infection.

#### RANTES

The level of RANTES was significantly increased with increasing time points post infection. At 3 h post infection (pi) the RANTES level was increased to 348% (*p* < 0.0001) which increased significantly at 6 h (466%, *p* < 0.0001), 12 h (697%, *p* < 0.0001) and reached to the level 1221% (*p* < 0.0001) at 24 h post infection (Fig. 6) when compared to the level expressed at 0 h post JEV infection. A significant 134%, *p* < 0.0001 increase in RANTES level at 6 h was noticed compared to 3 h post JEV infection. The level increase significantly to 150%, *p* < 0.0001 at 12 h compared to 6 h post infection. At 24 h the level was significantly increased by 175%, *p* < 0.0001 compared to 12 h post infection. Overall we noticed an increase in RANTES level with increasing time points post infection. The cytopathic effect was observed in Neuro2A cells at 12 h and 24 h post infection.

## Discussion

Microglia is the primary components of the CNS and plays critical role in innate immune system. Microglia produces cytokines and monitors the integrity of CNS. Activated microglia respond to inflammatory signals by modifying their gene expression at the transcription level and releasing cytokines that assist in recruiting leukocytes to the CNS [22].

Microglial activation is the central player in the CNS vulnerability following JEV infection which leads to increased proinflammatory mediators and peripheral immune cell infiltration into the brain. Mouse microglia is productively infected by JEV in vitro and is proposed to play a role in long-lasting infection.

Study by Ghoshal et al. showed that supernatant from JEV-infected microglia cell culture is capable of inducing neuronal cell death [9]. In this context, pro-inflammatory mediators such as TNF-α, IL1β, IL-6, and MCP-1 (CCL-2) secreted by activated microglia are responsible for neuronal death under JEV infection. Infection of BV2 cells with JEV resulted in significant higher expression of IL-6, TNF-α, MCP-1 and IL-1β as compared to un-infected cells [23]. In vitro neutralization of TNF-α derived from JEV-infected murine microglia cultures also reduces cytotoxicity to neuronal cells [13]. In vitro neutralization of TNF-α inhibits the production of CCL5 from JEV-infected glia. As a result, the latter supernatants show reduced chemotactic activity towards murine macrophages [24]. In mice, it is known that neuronal inflammation and neuronal death associated with JEV pathogenesis strongly depends on the NLRP3 inflammasome and the resulting maturation of pro-IL-1β and pro-IL-18 into IL-1β and IL-18 [25].

A critical role for IL-17, a cytokine produced by T helper 17 (Th17) cells, has been indicated in the pathogenesis of chronic inflammatory and autoimmune diseases. IL-17 signaling induces the production of proinflammatory cytokines (IL-1, IL-6, G-CSF, GM-CSF, and TNF) and chemokines (CXCL1, CXCL2, CXCL5, CCL2, CCL7, CCL20, and IL-8), matrix metalloproteinases (MMP1, MMP3, MMP9, and MMP13), and anti-microbial peptides (β-defensins, S-100 proteins) [26]. The role of IL-17 is well studied in ischemic brain injury. Increased expression of IL-17 mRNA in peripheral blood mononuclear cells was detected in patients after ischemic stroke. Also, higher expression of IL-17 at the mRNA and protein levels has been detected in the penumbral brain tissue 1, 3, and 6 days after reperfusion in mice [27]. In an animal model of ischemic stroke, an increased number of IL-17-producing blood mononuclear cells were observed [28]. In another study, IL-17 levels were elevated 3 days after reperfusion. Levels of inflammatory cytokines IL-1β, TNF-α, and matrix metalloproteinases, indicators of blood brain barrier (BBB) damage are decreased after stroke induction in IL-17-deficient mice [29]. Increased expression of IL-17 receptor on neurons has been shown simultaneously with increased expression of IL-17 in CNS tissue after stroke induction, indicating the role of IL-17 in direct neuronal damage [30]. IL-17 also enhances autophagy in neurons and thus aggravates neuronal ischemic injuries [31]. In a mouse model of dengue infection, IL-17 receptor-deficient mice infected with DENV-2 showed reduced lethality compared to wild-type (WT) mice, and treatment of DENV-2 infected mice with anti-IL-17 antibodies was associated with reduced disease severity [32]. Detrimental role of IL-17 was also noticed in COVID-19 infection [33].

We noticed upregulated mRNA expression of IL-17 and IL-6 in both BV2 and Neuro 2A cell lines. IL-17A has been reported to mediate neutrophil recruitment, activation, and migration [34]. Increased IL-17 expression within the CNS may contribute to the pronounced inflammation and severity of disease. IL-17 could enhance BBB damage by the disruption of tight junctions [35]and by the promotion of monocyte migration across the BBB through an intracellular adhesion molecule (ICAM) 1-dependent mechanism [36]. These results, together with those obtained from our study; strongly suggest that IL-17 production is correlated with JEV severity infections.

Interleukin-6 (IL-6) is an important cytokine mediator released in many immunological and inflammatory responses [37]. IL-6 is considered one of the most important cytokines during an infection, along with IL-1 and TNF-α. IL-6 affects the secretion of IFN-γ. In vivo blockage of IL-6 using a monoclonal antibody during acute infection in mice with murine leukemia virus resulted in reduced viral loads, and increased production of IFN-γ and the serine protease granzyme B (essential to produce apoptosis in target cells) [38].

We noticed increased levels of IL-6 at later time points post infection in both BV2 and Neuro2A cell line. In contrast the levels of IFN-γ were started decreasing with increasing time points post JEV infection in both BV2 and Neuro2A cell line. Increased levels of IL-6 might be exacerbating the tissue pathology during later infections by increasing inflammation followed by cytokine secretion and cellular recruitment.

Studies have been documented suggesting that the synergistic interaction between IL-6 and interleukin 17 (IL-17) have been associated with viral persistence and exacerbated clinical outcome during infection with Theiler's murine encephalomyelitis virus (TMEV). Genetically engineered mice carrying a human IL-6 transgene have excessive production of IL-6, leading to increased production of Th17 cells during an immune response. The IL-6 and IL-17 synergistic interaction leads to induction of anti-apoptotic molecules (Bcl-2 and Bcl-xL) inhibiting the destruction of TMEV-infected cells by virus-specific CD8 + T-cells, therefore favoring virus survival [39].

We noticed a decreased mRNA expression of IL-21 cytokine and FoxP3^+^with increasing time points post JEV infection in both BV2 and Neuro2A cell line. IL-21 is predominantly produced from CD4^+^T cells. It has been reported that IL-21 acts as a pro-inflammatory agent during multiple sclerosis progression and so it has been suggested that IL-21 may promote disease advancement [40]. Moreover, increased infiltration of pro-inflammatory cells into the CNS has also been seen when IL-21 was suppressed [41]. Another study demonstrated that IL-21 plays an anti-inflammatory role in LPS-induced macrophages [42]. The expression of cytokine and chemokine mRNA was remarkably increased in macrophages after LPS challenge and IL-21 was able to inhibit the expression of TNF-*α*, IL-6, and IL-1*β* at 3 h, 6 h, and 24 h in LPS-induced macrophages [42]. Furthermore, IL-21 inhibited the gene and protein expression of TLR4 and iNOS in LPS-stimulated macrophages [42].Studies have shown that IL-21 could affect the homeostasis of Tregs. The frequency of Treg cells and expressions of FoxP3^+^ were significantly decreased in both blocked IL-21 and IL-21R deficient mice with autoimmune encephalomyelitis (EAE) [41, 43]. FoxP3^+^ Treg cells exert vital functions involved in down-regulating the pathological T cell response through secretion of the anti-inflammatory cytokines IL-10 and TGF-β [44]. Our study on cell culture model is corroborated with above finding in which we reported decreased levels of IL-21 and FoxP3^+^ which suggests that FoxP3^+^ level was decreased due to decreased level of IL-21 suggesting IL-21 has a role in the homeostasis of Tregs in viral encephalitis. Also, we may conclude that release of IL-21 might have anti-inflammatory role in JEV infection. Insufficient release of IL-21 in our study of JEV infection of BV2 and Neuro 2A cell line might responsible for enhanced production of IL-6 and IL-17 cytokines. Detailed research is necessary to clarify the role of IL-21 in JEV infection using IL-21 blockade/inhibitor.

MCP-1 (CCL2) is a chemokine expressed by glial cells and neurons, and involved in the recruitment of astrocytes, microglia and infiltrating cells from the blood. The neuroinflammation caused by JEV is mainly related to the loss of control of microglia, which release inflammation-related cytokines and chemokines such as IL-1β, IL-6, TNFα, and MCP1, causing an irreversible inflammatory response and leading to neuronal necrosis. Supernatants from JEV-infected cultures showed a significant increase in RANTES production in a time-dependent manner [11]. JEV is able to modulate the production of inflammatory chemokines in human microglia in a dose-dependentmanner [45]. At the CNS level, CSF of JEV-infected mice present elevated levels of CCL2 [46]. Neutralization of CCL2 produced by JEV-infected murine glia reduces attraction of murine monocytes/macrophages cell line *in vitro* [11]. In the brain, mRNA levels of CCL2 in addition to other chemokines are higher in JEV-infected mice than in control mice [12]. Monocytes and granulocytes accumulate in the brain of JEV infected CCL2-deficient mice [47]. We noticed upregulated levels of MCP1 and RANTES in BV2 and Neuro 2A following JEV infection. Upregulated levels of these chemokines might be responsible for recruitment and infiltration of other cytokines/chemokines at the site of tissue injury.

We noticed a robust increase in TLR 2, 4 and 9 expression in both BV2 and Neuro 2A cell lines at later time point post infection. This result suggests that TLRs have a critical role in immunopathogenesis of JEV infection. Studies have shown that TLR4^−/−^ mice showed mild CNS inflammation manifested by reduced viral burden, leukocyte infiltration and proinflammatory cytokine expression. Interestingly, TLR4 ablation provided potent in vivo systemic type I IFN innate response, as well as ex vivo type I IFN production associated with strong induction of antiviral PRRs (RIG-I, MDA5), transcription factors (IRF-3, IRF-7), and IFN-dependent (PKR, Oas1, Mx) and independent ISGs (ISG49, ISG54, ISG56) by alternative activation of IRF3 and NF-κB in myeloid-derived DCs and macrophages [48]. This suggests that absence of TLR4 were closely coupled with reduced JE lethality. Additional studies are required to determine the detailed function of TLRs, cytokines and chemokines (which we studied in this paper) in order to understand the mechanisms how these immune mediators modulate disease status to provide future therapeutic targets/methods in JEV infection. This will help to prevent severe inflammation and death from JEV infection.

## Data Availability

The data obtained from the present study was statistically analyzed and presented in the result section. All the raw data generated from the study and materials will be made available on request by corresponding author.
